# Supplementary material for: A Critical View of the Application of the APEX Software (Aqueous Photochemistry of Environmentally-Occurring Xenobiotics) to Predict Photoreaction Kinetics in Surface Freshwaters
Source: Molecules. 2019 Dec 18;25(1):9. doi: 10.3390/molecules25010009 (PMC7017383; doi:10.3390/molecules25010009)
Supplement: Supplementary file 1 [file molecules-25-00009-s001.zip › 3.2.4_gcc-4.4.0.zip!/3.2.4_gcc-4.4.0/3.2.4_gcc-4.4.0/doc/octave/html/interpreter/Set-Operations.html]

Set Operations - Untitled


Up: Sets


---

### 26.1 Set Operations

Octave supports the basic set operations. That is, Octave can compute
the union, intersection, complement, and difference of two sets.
Octave also supports the *Exclusive Or* set operation, and
membership determination. The functions for set operations all work in
pretty much the same way. As an example, assume that `x` and
`y` contains two sets, then

```
     union(x, y)
```

computes the union of the two sets.

— Function File: [tf = **ismember** (A, S)  
— Function File: [tf, S\_idx] = **ismember** (A, S)  
— Function File: [tf, S\_idx] = **ismember** (A, S, "rows")  
> Return a matrix tf with the same shape as A which has a 1 if
> `A(i,j)` is in S and 0 if it is not. If a second output argument
> is requested, the index into S of each of the matching elements is
> also returned.
>
> ```
>           a = [3, 10, 1];
>           s = [0:9];
>           [tf, s_idx] = ismember (a, s);
>                => tf = [1, 0, 1]
>                => s_idx = [4, 0, 2]
> ```
>
> The inputs, A and S, may also be cell arrays.
>
> ```
>           a = {'abc'};
>           s = {'abc', 'def'};
>           [tf, s_idx] = ismember (a, s);
>                => tf = [1, 0]
>                => s_idx = [1, 0]
> ```
>
> With the optional third argument `"rows"`, and matrices
> A and S with the same number of columns, compare rows in
> A with the rows in S.
>
> ```
>           a = [1:3; 5:7; 4:6];
>           s = [0:2; 1:3; 2:4; 3:5; 4:6];
>           [tf, s_idx] = ismember(a, s, 'rows');
>                => tf = logical ([1; 0; 1])
>                => s_idx = [2; 0; 5];
> ```
>
>
>
>
> **See also:** unique, union, intersect, setxor, setdiff.

— Function File: **union** (a, b)  
— Function File: **union** (a, b, "rows")  
> Return the set of elements that are in either of the sets a and
> b. For example,
>
> ```
>           union ([1, 2, 4], [2, 3, 5])
>                => [1, 2, 3, 4, 5]
> ```
>
> If the optional third input argument is the string "rows" each row of
> the matrices a and b will be considered an element of sets.
> For example,
>
> ```
>           union([1, 2; 2, 3], [1, 2; 3, 4], "rows")
>                =>  1   2
>               2   3
>               3   4
> ```
>
> — Function File: [c, ia, ib] = **union** (a, b)  
> > Return index vectors ia and ib such that `a == c(ia)` and
> > `b == c(ib)`.
> >
> >
> >
> > **See also:** intersect, complement, unique.

— Function File: **intersect** (a, b)  
— Function File: [c, ia, ib] = **intersect** (a, b)  
> Return the elements in both a and b, sorted in ascending
> order. If a and b are both column vectors return a column
> vector, otherwise return a row vector.
>
> Return index vectors ia and ib such that `a(ia)==c` and
> `b(ib)==c`.


**See also:** unique, union, setxor, setdiff, ismember.

— Function File: **complement** (x, y)  
> Return the elements of set y that are not in set x. For
> example,
>
> ```
>           complement ([ 1, 2, 3 ], [ 2, 3, 5 ])
>                => 5
> ```
>
>
>
>
> **See also:** union, intersect, unique.

— Function File: **setdiff** (a, b)  
— Function File: **setdiff** (a, b, "rows")  
— Function File: [c, i] = **setdiff** (a, b)  
> Return the elements in a that are not in b, sorted in
> ascending order. If a and b are both column vectors
> return a column vector, otherwise return a row vector.
>
> Given the optional third argument "rows", return the rows in
> a that are not in b, sorted in ascending order by rows.
>
> If requested, return i such that `c = a(i)`.
>
>
>
> **See also:** unique, union, intersect, setxor, ismember.

— Function File: **setxor** (a, b)  
— Function File: **setxor** (a, b, 'rows')  
> Return the elements exclusive to a or b, sorted in ascending
> order. If a and b are both column vectors return a column
> vector, otherwise return a row vector.
> — Function File: [c, ia, ib] = **setxor** (a, b)  
> > Return index vectors ia and ib such that `a == c(ia)` and
> > `b == c(ib)`.
> >
> >
> >
> > **See also:** unique, union, intersect, setdiff, ismember.
